# Can physicians and schools mitigate social inequalities in human papillomavirus vaccine awareness, uptake and vaccination intention among adolescents? A cross-sectional study, France, 2021 to 2022

**DOI:** 10.2807/1560-7917.ES.2023.28.46.2300166

**Published:** 2023-11-16

**Authors:** Hadrien Moffroid, Damien Oudin Doglioni, Sandra Chyderiotis, Jonathan Sicsic, Anne-Sophie Barret, Jocelyn Raude, Sebastien Bruel, Aurelie Gauchet, Morgane Michel, Amandine Gagneux-Brunon, Nathalie Thilly, Judith E Mueller, on behalf of the PrevHPV Consortium

**Affiliations:** 1 Institut Pasteur, Université Paris Cité, Emerging Disease Epidemiology Unit, 75015 Paris, France; 2 University of Melbourne, Melbourne, Australia; 3 Université Paris Cité, LIRAES, 75006, Paris, France; 4 Santé Publique France, St Maurice, France; 5 Université Rennes, EHESP, CNRS, Inserm, Arènes - UMR 6051, RSMS (Recherche sur les Services et Management en Santé) - U1309 – 35000 Rennes, France; 6 Department of General Practice, Faculté de Médecine Jacques Lisfranc, Université Jean Monnet, Université de Lyon, Saint-Etienne, France; 7 Health, Systemic, Process UR 4129 Research Unit, University Claude Bernard, University of Lyon, Lyon, France; 8 Université Savoie Mont Blanc, Université Grenoble Alpes LIP/PC2S, Grenoble, France; 9 Université Paris Cité, ECEVE, UMR1123, Inserm, Paris, France; 10 Assistance Publique-Hôpitaux de Paris, Hôpital Robert Debré, Unité d’épidémiologie clinique, Paris, France; 11 CHU de Saint-Etienne – Service d’infectiologie; 12 Centre International de Recherche en Infectiologie, Team GIMAP, Université de Lyon, Université Jean Monnet, Université Claude Bernard Lyon 1, Inserm, U1111, CNRS, UMR530; 13 Université de Lorraine, APEMAC, Nancy, France; 14 Université de Lorraine, CHRU-Nancy, Département Méthodologie, Promotion, Investigation, Nancy, France; 15 Members of the PrevHPV Consortium are listed under acknowledgements

**Keywords:** HPV vaccination, uptake, acceptance, awareness, social inequalities, general practitioner, school-based initiatives, France

## Abstract

**Background:**

In France, human papillomavirus (HPV) vaccination coverage varies across socioeconomic levels.

**Aim:**

We aimed at assessing HPV vaccine awareness, uptake and vaccination intention among adolescents in France.

**Methods:**

In a cluster-randomised study, 13–15-year-old students in 61 French middle schools completed a web-based questionnaire. We used multivariable logistic regression to evaluate determinants of HPV vaccine awareness, self-reported uptake and vaccination intention among unvaccinated students and interaction terms to explore effects of visits to family physician and remembering school lessons on vaccination. The French deprivation index of school municipalities served as proxy for socioeconomic levels.

**Results:**

Among 6,992 participants, awareness was significantly associated with parental education (odds ratio (OR) = 0.82; 95% confidence interval (CI): 0.71–0.95), language spoken at home (OR = 0.59; 95% CI: 0.52–0.66) and deprivation level (OR = 0.57; 95% CI: 0.44–0.71), regardless of physician visit or school lessons. Vaccine uptake was associated with parental education without a recent physician visit (OR = 0.31; 95% CI: 0.16–0.59, vs OR = 0.64; 95% CI: 0.52–0.78 with a visit, interaction p = 0.045). Vaccination intention among unvaccinated was associated with deprivation level (moderate-low vs low) among students not remembering school lessons on vaccination (OR = 0.17; 95% CI: 0.05-0.62, vs OR = 0.93; 95% CI: 0.51–1.67 remembering school lessons, interaction p = 0.022). Parental education was associated with vaccination intention among students reporting a physician visit (OR = 0.41; 95% CI: 0.26–0.64 vs OR = 1.05; 95% CI: 0.50–2.20 without a visit, interaction p = 0.034).

**Conclusion:**

Our results suggest that healthcare and school could promote vaccination and mitigate social inequalities in HPV vaccination coverage.

**Key public health message**

**What did you want to address in this study?**

Human papillomavirus (HPV) can cause genital cancer. Vaccination against HPV protects against virus types causing most cases of cervical cancer, as well as some other cancers. Vaccine uptake among adolescents varies in France and other European countries. We wanted to investigate awareness, uptake and intention of HPV vaccination among French adolescents and factors affecting vaccination.

**What have we learnt from this study?**

Adolescents were less aware of vaccination and less often vaccinated or planning to vaccinate if their parents had a lower education, other languages than French were spoken at home and the family lived in a poorer area. Importantly, if the adolescents had recently visited a family physician or were taught about vaccinations at school, vaccination or planning to vaccinate were independent of parental education, language or neighbourhood.

**What are the implications of your findings for public health?**

Family physicians and school lessons on vaccination are important in promoting systematic vaccination of all adolescents against HPV, irrespective of their social context. How to use such communication ways to enhance vaccination in France needs further investigation.

## Introduction

Human papillomavirus (HPV) vaccines are highly effective against cervical and other HPV-related cancers, while being safe and generating population immunity [1]. In France, HPV vaccination has been recommended to girls since 2007, with an extension to boys since 2021 [2]. The target age is 11–14 years with catch-up up to 19 years. Despite a recent increase in HPV coverage estimates, immunisation coverage in France remains among the lowest within Europe [3]. By the end of 2021, coverage of the first dose among 15-year-old girls was 45.8% and that of the second dose among 16-year-old girls was 37.4%, while coverage of the first dose among 15-year-old boys was 6% [4].

Previous studies suggest that the main barriers to HPV vaccination in France are lack of parental awareness and low perceived accessibility, combined with widespread doubts on the need and safety of the vaccine [5]. In particular, there is evidence that general practitioners (GP) do not systematically offer or recommend this vaccine to adolescents [6]. Vaccination against HPV is today available at the offices of physicians (mainly GPs) and midwives. Finally, although the HPV vaccine is entirely reimbursed for most families in France by the combination of national and complementary health insurance and can be accessed free-of-charge in certain health centres, the access is complicated by the exclusive distribution through pharmacies.

As in other countries [7], HPV vaccine coverage in France shows a substantial gradient across socioeconomic groups [4,8]. It is unclear whether these inequalities arise from lower offer and access or lower acceptance in specific groups. Thus, characterising inequalities not only in vaccine uptake, but also in awareness and intention to get vaccinated, as well as identifying mitigating factors, appears necessary.

A systematic review suggested that young adolescents, the target group for HPV vaccination in most countries, have relatively favourable attitudes towards HPV vaccination, but lack awareness [9]. To date, minors in France need parental consent for vaccination, however, increasing evidence emphasises the integral role adolescents have in the decision-making process on HPV vaccination [10]. Little evidence is available on social inequalities around HPV vaccine awareness and intention to get vaccinated among young adolescents in Europe, but one study in Italy described better HPV knowledge among adolescents with higher level of parental education [11].

In this context, we aimed to delineate socio-educational inequalities of HPV vaccine awareness, self-reported uptake and vaccination intention among French adolescents aged 13–15 years, and to investigate whether visits to the family GP and school lessons on vaccination could mitigate these inequalities.

## Methods

### Participant recruitment

The national research programme to improve HPV vaccine coverage among French adolescents, PrevHPV, is a cluster-randomised study in mainland France evaluating the effect of a multicomponent intervention on HPV vaccine uptake among adolescents irrespective of their sex [12]. Between 22 November 2021 and February 2022, we collected data from students in French middle schools via an anonymous web-based questionnaire published on the REDcap tool (https://www.project-redcap.org/). Middle schools in nine regions throughout the French mainland were randomised as previously described [12]. Ninety-one schools were invited to participate in the survey. A total of 19,885 third and fourth grade students, typically aged 13–15 years, were eligible to participate. No written consent was required for an anonymous survey in France, but all participants were informed of their right not to participate. After assenting to participate, the adolescents completed the questionnaires during in-class lessons, under the supervision of their biology teacher or school nurse. Parents received information about the study and could decline the participation.

### Questionnaires, data collection and management

The questionnaire included questions on socio-educational characteristics of the adolescents’ family, awareness, knowledge, perceptions and behaviour around HPV-related disease and vaccination, their self-declared HPV vaccination status and intention to get vaccinated. We assessed intention to get vaccinated among unvaccinated adolescents aware of HPV vaccine as follows: refusal (‘HPV vaccination is not relevant for me’), indecision (‘I consider HPV vaccination as relevant for me, but I am not sure about getting vaccinated’) or intention (‘I have the intention to soon get vaccinated’). More details can be seen in Supplementary Table 2.

To assess the socio-educational characteristics, we collected individual and ecological variables. Educational level of the mother and the father was grouped as ’below or equal to high school’, ‘above high school’ and ‘do not know’. We assessed language by the question ‘Do you commonly speak another language than French at home’ (French or other languages). For specific analyses, we collated parental education (the highest achieved level among parents) and language. We assumed that the relevance of a multilingual family environment for vaccine uptake depends on the level of parental education.

As an ecological study, we used the 2015 French deprivation index (Fdep) [13], a proxy measure for an area-based socioeconomic level and disparity. The Fdep is based on the median household income, the percentage of high school graduates in the population of persons aged 15 years or over, the proportion of working class in the active population and the unemployment rate, with a mean of 0 used for mainland France. The municipalities of the participating schools had an index spanning from -2.2 to 2.2, which represents approximately the range of the index in French municipalities. Using this index, we created four categories of local deprivation level: low (least deprived: index ≤ -1), moderate–low (index > -1 to 0), moderate–high (index > 0 to 1), and high (most deprived: index > 1).

Knowledge on health-related subjects covered at school was assessed as remembering specific topics taught during school lessons (bacteria and viruses, vaccination in general; human reproduction, sexual education and sexually transmitted infections). In France, these topics can be addressed in biology classes in middle schools, but the content or the format is not harmonised. We assessed whether the students had seen their GP during the past 12 months and whether the GP had offered HPV vaccination. In the French context, the gatekeeper function of family physicians comprises mostly GPs. Although other medical specialities (e.g. paediatricians) can take this role, for simplicity, we refer here to GPs. Adolescents commonly visit GPs for acute or chronic health issues and for medical certificates of fitness to participate in sports. Three GP visits dedicated to health promotion, including vaccination, are fully reimbursed for children between the ages of 8 and 16 years. For specific analyses, we collated these variables into one variable (no visit/visit with a vaccine offer/visit without a vaccine offer). Most questions included a ‘do not know’ modality, which was included into ‘no’. More information can be seen in Supplementary Table SM1.

Additionally, we collected variables not part of the main hypothesis but known to impact health behaviour [14]. These included health-related behaviour and aptitudes evaluated as: self-efficacy (confidence in being able to respond to questions on one’s health, rated on a 10-point scale), ease of finding information on HPV and ease of talking to health professionals and close persons about HPV and social influence variables, on the attitudes of family and the social environment towards vaccinations in general and to HPV, respectively and the HPV vaccination status of friends. We assessed specific knowledge and attitude items on HPV using a 5-point Likert scale, coding responses as disagree/undecided/agree.

### Data analysis

We performed descriptive analyses on socio-educational characteristics by sex and on HPV vaccine knowledge and attitude items between subgroups according to socio-educational and mitigation variables. We used chi squared tests for differences between subgroups.

For the first analysis on determinants of HPV vaccine awareness (binary), vaccine status (binary) and vaccination intention (three levels: refusal, indecision, intention), we built multivariable logistic and multinomial regression models including all socio-educational variables and variables related to contact with GP, health-related knowledge, behaviour and aptitudes and social influence. For each outcome, we included in the final multivariable model only variables associated in bivariable models at p value < 0.2.

For the main objective of exploring social inequalities, we analysed, for each outcome, a model including only socio-educational variables (grade level, sex, parental education, language, deprivation level). We then explored mitigation including interaction terms between socio-educational determinants (parental education, language, deprivation level) and hypothetical mitigation variables (health-related knowledge and recent GP visit). Interaction terms with p < 0.05 were interpreted as mitigation, and in this case, the effects of determinants were represented stratifying for the mitigation factor.

Statistical significance was defined as p value < 0.05. Data analyses were performed on Stata Version 17 (StataCorp, the United States).

## Results

### Participant characteristics

Given constraints during the COVID-19 pandemic, only 61 middle schools with 12,833 students in third and fourth grades participated in the survey. While no information on the number of school classes was collected, we estimated that baseline questionnaires were administered in 70% of classes. More details can be seen in Supplementary Figure SM2.

In total, 7,632 persons opened the questionnaire, with 7,580 accepting participation and 6,992 leaving valid responses, or 54.5% (6,992/12,833) of the eligible students. There were 3,564 females and 3,428 males responding, approximately equally distributed over the two school grade levels and with similar socio-educational characteristics. More information can be seen in Supplementary Table SM3. Almost half of the participants were unaware of at least one parent’s educational level, while 32.3% and 25.4%, respectively, reported higher education for their mothers and fathers. Other languages than French were spoken at home of 1,480 (21.2%) respondents. Of these, 335 (4.8%) had parents with lower education and 541 (7.7%) had higher education. A total of 611 (8.7%) participants attended school in a low and 1,310 (18.7%) in a high deprivation area. Overall, 1,233 (17.6%) 24.1% participants reported no recent GP visit (more frequent among males and those with lower parental education) and 35.7% reported a visit without an offer to vaccinate against HPV (more frequent among males). More information can be seen in Supplementary Table SM4. Similarly, 70.3% remembered school lessons on vaccination.

Overall, 4,051 (57.9%) participants had heard about the HPV vaccine (75.9% of females, 51.6% of males) and 421 (6.0%) were not sure. More information can be seen in Supplementary Figures SM5 and SM6. Among the participants aware of HPV vaccine or not sure but reporting their vaccination status (n = 4,385, information missing from 87 respondents), 1,568 (35.8%) knew they were vaccinated (48.0% of females, 17.0% of males), while 2,344 (53.5%) were not and 473 (10.8%) were not sure, seen in Supplementary Figures SM5 and SM7. Among the 2,815 participants aware of the HPV vaccine but not vaccinated or not sure of vaccination, 939 (33.4%) intended to get vaccinated (38.1% of females, 28.7% of males) and 311 (11.1%) did not, while 1,565 (55.6%) were not sure, seen in Supplementary Figures SM5 and SM8. In summary, among the 4,385 participants aware of the HPV vaccine, 2,507 (57.2%; 67.7% of females and 40.8% of males) were either vaccinated or intended to get vaccinated.

### Awareness of human papillomavirus vaccine

**Figure 1:**
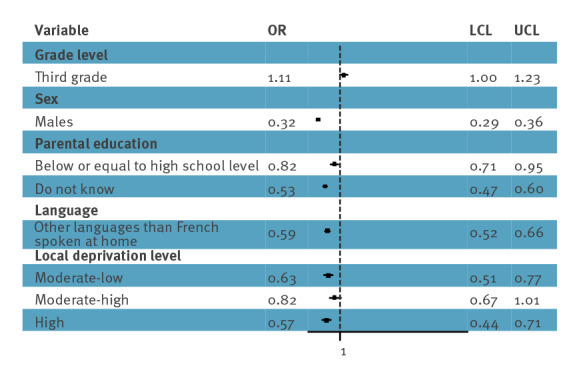
Awareness of human papillomavirus vaccine among middle school students, France, 2021–22 (n = 6,992)

LCL: lower 95% confidence limit; OR: odds ratio; UCL: upper 95% confidence limit.

In general multivariable analyses, awareness of HPV vaccine was significantly associated with male sex (odds ratio (OR) = 0.32; 95% confidence interval (CI): 0.29–0.36), language spoken at home (OR = 0.37–0.50, depending on the level of parental education), high local deprivation (OR = 0.56; 95% CI: 0.44–0.71), high self-efficacy (OR = 1.84; 95% CI:1.36–2.50, a GP visit (OR = 1.60; 95% CI: 1.39–1.85) and knowledge on vaccination (OR = 1.51; 95% CI: 1.34–1.72) or on sexually transmitted infections (OR = 1.50; 95% CI: 1.32–1.72) (Table). More details can be seen in Supplementary Table 6 **.** When only variables grade level, sex, parental education, language and deprivation level were included in the model, the respondents were less aware of the HPV vaccine if they lived in an area with high local deprivation (OR = 0.57; 95% CI: 0.44–0.71), spoke other languages than French at home (OR = 0.59; 95% CI: 0.52–0.66) and had parents with a lower level of education (OR = 0.82; 95% CI: 0.71–0.95) (Figure 1). Visits to the GP or school lessons did not increase awareness in these respondents.

**Table:** Awareness, uptake and intention of human papillomavirus vaccination among middle school students, France, 2021–22 (n = 6,992)^a^

Characteristics	Awareness (n = 6,992)					Vaccine uptake among persons aware of HPV vaccination (n = 4,385)						Vaccination intention among unvaccinated (n = 2,815)					
	n	%	OR	95% CI	p value	n	%	OR	95% CI		p value	n	%	OR	95% CI		p value
**School grade**																	
Fourth	3,742	53.5	1	NA		2,271	51.8	1	NA			1,521	54.0	1	NA		
Third	3,250	46.5	0.98	0.86–1.10	0.704	2,114	48.2	1.24	1.02–1.50		0.032	1,294	46.0	1.17	0.83–1.66		0.372
**Sex**																	
Female	3,564	51.0	1	NA		2,665	60.8	1	NA			1,388	49.3	1	NA		
Male	3,428	49.0	0.32	0.29–0.36	< 0.001	1,720	39.2	0.42	0.35–0.51		< 0.001	1,427	50.7	0.47	0.34–0.66		< 0.001
**Parental education level and languages spoken at home**																	
French																	
Below or equal to high school	1,122	16.1	0.87	0.73–1.04	0.119	760	17.3	0.74	0.57–0.96		0.022	510	18.1	0.68	0.41–1.13		0.134
Above high school	2,198	31.4	1	NA		1,597	36.4	1	NA			889	31.6	1	NA		
Do not know	2,192	31.4	0.56	0.48–0.65	< 0.001	1,235	28.2	0.97	0.77–1.22		0.797	841	29.9	0.49	0.31–0.78		0.002
Other languages																	
Below or equal to high school	335	4.8	0.50	0.38–0.65	< 0.001	185	4.2	0.56	0.33–0.92		0.024	139	4.9	0.64		0.31–1.35	0.242
Above high school	541	7.7	0.47	0.38–0.59	< 0.001	309	7.1	1.02	0.71–1.48		0.899	206	7.3	0.35		0.18–0.68	0.002
Do not know	604	8.6	0.37	0.30–0.46	< 0.001	299	6.8	0.68	0.46–1.01		0.059	230	8.2	0.31		0.16–0.59	< 0.001
**Local deprivation index**																	
Number of responses	6,903					4,333						2,782					
Low	611	8.9	1	NA		438	10.1	1	NA			257	9.2	1		NA	
Moderate-low	2,462	35.7	0.62	0.50–0.77	< 0.001	1,474	34.0	0.84	0.61–1.12		0.275	949	34.1	0.72		0.38–1.37	0.317
Moderate-high	2,520	36.5	0.84	0.67–1.04	0.116	1,669	38.5	0.75	0.55–1.03		0.090	1,090	39.2	0.88		0.46–1.66	0.692
High	1,310	19.0	0.56	0.44–0.71	< 0.001	752	17.4	1.03	0.72–1.47		0.883	486	17.5	0.49		0.25–0.98	0.042
**Health-related behaviour and aptitudes**																	
Self-efficacy^b^																	
0–2.5	247	3.5	1	NA		106	2.4	NA^c^				70	2.5	1		NA	
2.6–5.0	757	10.8	1.22	0.87–1.70	0.244	391	8.9					273	9.7	1.44		0.51–4.08	0.496
5.1–7.5	1,363	19.5	1.49	1.08–2.04	0.014	799	18.2					554	19.7	1.92		0.71–5.19	0.202
7.6–10	4,625	66.2	1.84	1.36–2.50	< 0.001	3,089	70.4					1,918	68.1	1.49		0.58–3.84	0.407
Ease to find information about HPV vaccination																	
Number of responses	NA					4,241						2,713					
Disagree	NA^b^					302	7.1	1	NA			215	7.9	1		NA	
Unsure						1,860	43.9	0.81	0.56–1.18		0.265	1,247	46.0	1.43		0.85–2.39	0.178
Agree						2,079	49.0	0.76	0.52–1.10		0.146	1,251	46.1	2.63		1.55–4.47	< 0.001
Difficult to talk to close family or friends about HPV vaccination																	
Agree	NA					592	13.5	1	NA			440	15.6	1		NA	
Unsure						1,100	25.1	1.28	0.92–1.78		0.149	802	28.5	0.89		0.51–1.54	0.680
Disagree						2,693	61.4	1.04	0.77–1.41		0.786	1,573	55.9	1.04		0.63–1.72	0.874
Difficult to talk to health professionals about HPV vaccination																	
Agree	NA					580	13.2	1	NA			412	14.6	1		NA	
Unsure						1,250	28.5	1.38	1.00–1.92		0.050	892	31.7	0.95		0.56–1.63	0.862
Disagree						2,555	58.3	1.21	0.90–1.64		0.199	1,511	53.7	1.21		0.73–1.99	0.464
GP visit during the past 12 months																	
Number of responses	6,861					NA											
No visit	1,233	18.0	1		NA	NA											
Do not remember	1,030	15.0	1.43	1.19–1.73	< 0.001												
Visited	4,598	67.0	1.60	1.39–1.85	< 0.001												
GP visit during the past 12 months and a vaccine offer																	
Number of responses	NA					4,382						2,812					
No visit	NA					1,002	22.9	1	NA			922	32.8	1	NA		
Visit, vaccine not offered						1,520	34.7	1.47	1.08–1.99		0.045	1,338	47.6	0.97	0.67–1.40		0.883
Visit, vaccine offered						1,860	42.5	19.09	14.38–25.34		< 0.001	552	19.6	3.21	1.91–5.39		< 0.001
General attitude to vaccination within the family																	
Unfavourable	705	10.1	1	NA		369	8.4	1	NA			291	10.3	1	NA		
Unsure	1,547	22.1	0.98	0.80–1.20	0.848	897	20.5	0.66	0.44–0.99		0.045	666	23.7	1.74	1.00–3.05		0.052
Favourable	4,740	67.8	1.24	1.03–1.50	0.020	3,119	71.1	0.90	0.62–1.29		0.562	1,858	66.0	3.66	2.20–6.08		< 0.001
Attitude to HPV vaccination in the social environment																	
Number of responses	NA					4,114						2,600					
Unfavourable	NA					427	10.4	1	NA			356	13.7	1		NA	
Unsure						1,342	32.6	1.80	1.24–2.62		0.002	1,057	40.7	7.28		4.41–12.01	< 0.001
Favourable						2,345	57.0	3.73	2.61–5.34		< 0.001	1,187	45.7	31.07		18.02–53.57	< 0.001
HPV vaccination status of friends																	
Number of responses	NA					4,306						2,762					
Not vaccinated	NA					446	10.4	1	NA			382	13.8	1		NA	
Do not know						1,642	38.1	1.78	1.23–2.60		0.002	1,270	46.0	1.90		1.20–3.01	0.006
Vaccinated						2,218	51.5	2.97	2.07–4.26		< 0.001	1,110	40.2	3.17		1.98–5.07	< 0.001
**Remembrance of school lessons**																	
Infections by bacteria or viruses																	
Number of responses	6,899					4,315						2,767					
No/do not know	2,482	36.0	1	NA		1,426	33.1	NA^c^				905	32.7	1		NA	
Yes	4,417	64.0	1.09	0.97–1.23	0.16	2,889	67.0					1,862	67.3	0.87		0.61–1.25	0.454
Vaccination																	
Number of responses	6,903					4,328						2,773					
No/do not know	1,989	28.8	1	NA		1,108	25.6	1		NA		685	24.7	1	NA		
Yes	4,914	71.2	1.51	1.34–1.72	< 0.001	3,220	74.4	0.87	0.71–1.08		0.204	2,088	75.3	0.94	0.59–1.49		0.789
Human reproduction (anatomy and physiology)																	
Number of responses	6,901					4,337						2,779					
No/do not know	1,135	16.5	1	NA		655	15.1	NA^c^				433	15.6	NA^c^			
Yes	5,766	83.6	0.88	0.74–1.04	0.14	3,682	84.9					2,346	84.4				
Sexual education on behavioural and social aspects																	
Number of responses	6,872					4,317						2,770					
No/do not know	2,834	41.2	1	NA		1,715	39.7	1			NA	1,131	40.8	NA^c^			
Yes	4,038	58.8	1.01	0.89–1.15	0.91	2,602	60.3	1.00	0.80–1.25		0.991	1,639	59.2				
Sexually transmitted infections																	
Number of responses	6,846					4,300						2,760					
No/do not know	2,231	32.6	1	NA		1,233	28.7	1			NA	821	29.8	NA^c^			
Yes	4,615	67.4	1.50	1.32–1.72	< 0.001	3,067	71.3	1.03	0.84–1.25		0.810	1,939	70.3				

CI: confidence interval; GP: general practitioner; HPV: human papillomavirus; NA: not applicable; OR: odds ratio.

^a^ In case the number of responses differs from the total number of respondents, the numbers are given by the variable.

^b^ Self-efficacy was evaluated on a 10-point scale.

^c^ Not included in the model because p ≥ 0.20 in bivariable analysis.

Multivariable models for the three outcomes human papillomavirus vaccine awareness, uptake and intention containing all variables that demonstrated a p-value < 0.2 in bivariable analyses.

Multivariable regression model including only socio-demographic characteristics. No significant interaction between a recent visit to the general practitioner or school lessons on vaccination was found.

### Uptake of human papillomavirus vaccine

**Figure 2:**
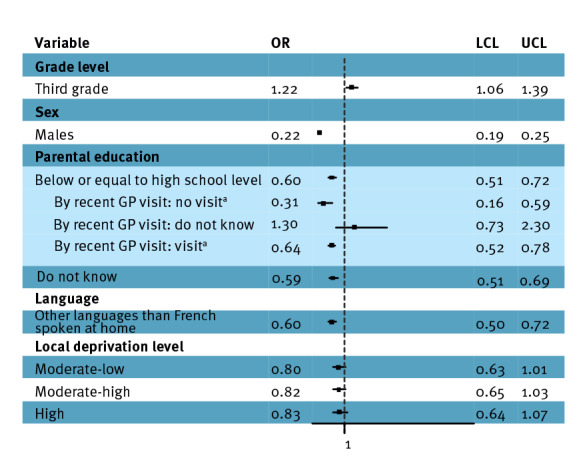
Uptake of human papillomavirus vaccine among middle school students aware of HPV vaccine and mitigation by physicians and school on vaccination, France, 2021–22 (n = 4,333)

GP: general practitioner; LCL: lower 95% confidence limit; OR: odds ratio; UCL: upper 95% confidence limit.

^a^ Interaction term p = 0.045.

Self-reported vaccine uptake among respondents aware of the vaccine was significantly associated with a GP visit with (OR = 19.09; 95% CI: 14.38–25.34) and without (OR = 1.47; 95% CI: 1.08–1.99) a vaccine offer, a social environment favourable to HPV vaccination (OR = 3.73; 95% CI: 2.61–5.34), friends being vaccinated against HPV (OR = 2.97; 95% CI: 2.07–4.26), male sex (OR = 0.42; 95% CI: 0.35–0.51) and fourth grade at school (instead or third) (OR = 1.24; 95% CI: 1.02–1.50) (Table). More information can be seen in Supplementary Table SM7. In the model focusing on socio-educational determinants, vaccine uptake was decreased when the parents had a lower level of education (OR = 0.60; 95% CI: 0.51–0.72) and other languages than French were spoken at home (OR = 0.60; 95% CI: 0.50–0.72) (Figure 2). Low level of parental education was significantly less strongly associated with vaccine uptake among participants who reported a recent GP visit (OR = 0.64; 95% CI: 0.52–0.78) than among those who did not (OR = 0.31; 95% CI: 0.16–0.59, interaction term p = 0.045).

Multivariable regression models including only socio-demographic characteristics. Subgroup strata are represented if interaction terms with a recent visit to a general practitioner and school lessons on vaccination showed a p value < 0.05.

### Intention to get vaccinated among unvaccinated participants

**Figure 3:**
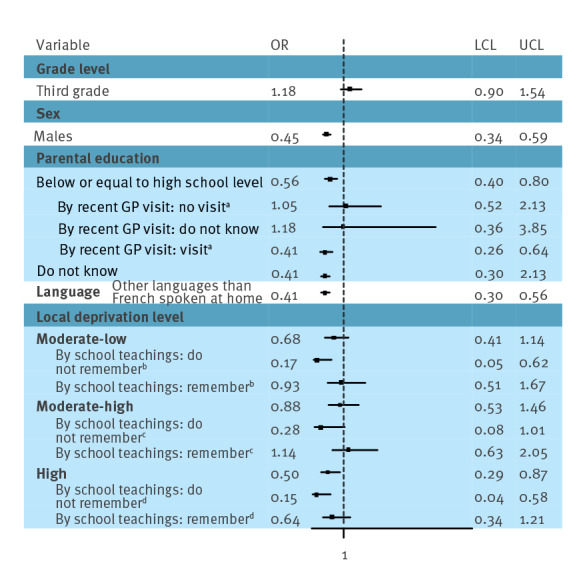
Intention to get vaccinated against human papillomavirus among unvaccinated middle school students, by recent visit to a general practitioner and school lessons on vaccination, France, 2021–22 (n = 2,782)

GP: general practitioner; LCL: lower confidence limit; OR: odds ratio; UCL: upper confidence limit.

^a^ Interaction term p value 0.034.

^b^ Interaction term p value 0.022.

^c^ Interaction term p value 0.053.

^d^ Interaction term p value 0.063.

The participants had significantly higher intention to get vaccinated if the attitudes towards HPV vaccination were more favourable in their social environment (OR = 31.07; 95% CI: 18.02–53.57) or in the family (OR = 3.66; 95% CI: 2.20–6.08), they had visited the GP and received a vaccine offer (OR = 3.21; 95% CI: 1.91–5.39), their friends were vaccinated (OR = 3.17; 95% CI: 1.98–5.07) or they considered finding of HPV-vaccine-related information easy (OR = 2.63; 95% CI: 1.55–4.47) (Table). Notably, males were 0.47 times (95% CI: 0.34–0.66) less likely intending to get vaccinated than females. In the model focusing on socio-educational determinants, the intention was decreased if other languages than French were spoken at home (OR = 0.41; 95% CI: 0.30–0.56), the local area was highly deprived (OR = 0.50; 95% CI: 0.29–0.87) or the parents had a lower level of education (OR = 0.56; 95% CI: 0.40–0.80) (Figure 3). The deprivation level of the school area was significantly associated with vaccine intention only among participants who did not remember school lessons on vaccination, but not among those remembering such lessons. This mitigation effect was significant (interaction term p = 0.022) for moderate-low deprivation vs low deprivation (OR = 0.17; 95% CI: 0.05–0.62 with remembering lesson vs. OR = 0.93; 95% CI: 0.51–1.67 not remembering). By contrast, the association between lower level of parental education and vaccination intention was found only among participants who reported a recent GP visit (OR = 0.41; 95% CI: 0.26–0.64, interaction term p = 0.034).

Multivariable regression model including only socio-demographic characteristics. Subgroup strata are represented if interaction terms with recent visit to the general practitioner and school lessons on vaccination showed a p value < 0.05.

### Knowledge and perceptions surrounding human papillomavirus vaccination

**Figure 4:**
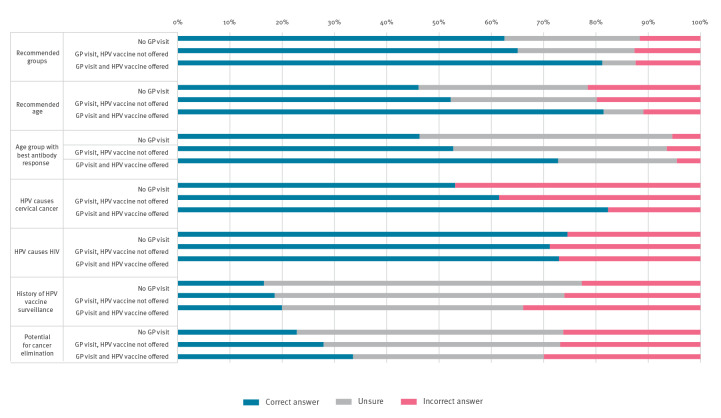
Perceptions and knowledge on human papillomavirus vaccination among middle school students aware of human papillomavirus vaccination, by visit to the general practitioner and offer of a vaccine in last 12 months, France, 2021–22 (n = 4,333)

Participants who had visited a GP and been offered a vaccine had better knowledge on HPV and HPV vaccine than those with no GP visit (Figure 4). For example, 5,697 (81.5%) participants with a GP visit and a vaccine offer had knowledge about the recommended age of HPV vaccination, 3,653 (52.3%) of those with a GP visit but not a vaccine offer had knowledge about the age and 3,223 (46.1%) persons with no GP visit had knowledge (p value < 0.001). Considering HPV vaccine as safe varied between these groups (46.7% with no GP visit, 54.5% with a GP visit but no vaccine offers and 76.3% with GP visit and a vaccine offer, p value < 0.001). Similarly, considering that HPV vaccine was easy to access varied (40.4%, 51.3% and 81.3%), respectively, p value < 0.001).

## Discussion

In this cross-sectional study among adolescents aged 13–15 years in France, we identified several socio-educational inequalities in HPV vaccine awareness, uptake and vaccination intention, relating to lower parental education, languages spoken at home and higher local deprivation levels. Recent GP visits mitigated inequalities in vaccine uptake and school lessons on vaccination mitigated inequalities in vaccination intention among unvaccinated adolescents. Surprisingly, for unvaccinated adolescents, we found disparities in vaccination intention by parental education specifically among those who reported a recent GP visit.

The use of other languages than French at home was associated with lower HPV vaccine awareness, uptake and vaccination intention, irrespective of parental education level. Following French research regulations on good ethical practice, we did not collect information on family origin, ethnicity or religious background, and consequently, we cannot fully interpret this disparity. However, given the independence from parental education level, the most relevant explanation may be language or cultural barriers leading to lower understanding of and lower feeling of being targeted by health promotion messages. This would be consistent with studies from the United States that have demonstrated the challenges faced by diverse communities regarding access to culturally and linguistically adapted HPV information [15,16]. These observations call for further exploration in the French context and possibly more targeted communication.

The fact that inequalities In HPV vaccine awareness were not mitigated by school lessons on vaccination or recent physician visit appears surprising. Further investigation is needed to explore the reasons behind such inequalities, including lower health literacy (capacity to understand the vaccine information) and selective information (to not mention HPV vaccines towards specific groups). To overcome inequalities in HPV vaccine awareness among adolescents, specific targeted interventions on HPV vaccine may be needed.

The prominent role of GPs in our study echoes previous research highlighting that limited access to GPs, GPs not offering the vaccine and challenges faced by GPs in promoting the HPV vaccine act as major barriers to HPV vaccination in France [6] and other countries [17,18]. These barriers include low adherence to routine consultations for adolescents, limited time during consultations, competing priorities for preventive interventions, extensive discussions needed with families regarding HPV vaccination, temptation to selectively offer vaccination to avoid refusal and optimise time, doubts about the vaccine usefulness, effectiveness and safety, as well as the sensitive nature of the association of the HPV vaccine with sexuality [6]. Our results suggest that these barriers contribute to social inequalities in vaccine awareness, uptake and vaccination intention. Various interventions, such as policies on adolescent routine consultations and vaccine provision, improved medical training and decision-aid tools, could address these difficulties. The PrevHPV project, for which our data were collected, includes an evaluation of the effectiveness of such decision-aid tool and communication training for GPs to improve practice and increase HPV vaccine uptake.

Knowledge and perceptions on HPV vaccine among teenagers have been well researched across Europe and the world [19,20], however, with little attention to social disparities. In this study, we describe significant disparities in knowledge and perceptions among adolescents based on social, economic and educational factors. There were large differences in knowledge and perception among participants depending on whether the adolescents had recently visited a physician. In the absence of comparable studies, such differences should be monitored in other European settings.

Adolescents’ HPV knowledge remains sub-optimal, with only 51.8% across 16 European countries [19]. Many young adolescents are not aware of the vaccine and lack support from healthcare providers, despite relatively positive attitudes [6,10]. In our study, participants showed more favourable knowledge and attitudes compared with previous studies involving adolescents in France [21] and the European Union [6,22]. Notably, only a small proportion of adolescents in our study expressed a negative perception of vaccine safety and both sexes had positive attitudes towards the utility and accessibility of vaccines.

In the analysis of wider determinants, the opinion of the social environment on HPV vaccination was the dominant determinant of vaccine intention among unvaccinated adolescents, concordant with the existing literature [23]. However, as many as 68% of female adolescents aware of the HPV vaccine were vaccinated or intended to get vaccinated – a coverage level that would allow significant public health benefit. Negative social influences may thus act as a major barrier to vaccination for one third of female adolescents and therefore should not be considered an insurmountable barrier to a successful vaccination programme. Appropriate interventions should address HPV vaccine attitudes in the wider population, and some studies showed a burgeoning success of social media campaigns in HPV health promotion [24,25], while peer-focused interventions may be particularly relevant for adolescents [26].

The mitigation of inequality by socioeconomic level in case of school lessons on vaccination was largely carried by an increase in intention in municipalities with higher deprivation levels, with little change in low-deprivation areas. Studies on free school-based HPV vaccine access in Belgium and in Canada have described a similar pattern, with such interventions benefiting higher-deprivation areas [27,28]. Thus, the development of vaccine access policies should be evidence-driven, as the benefits may be less tangible to the social environment of decision-makers. School-based interventions need careful design to avoid further increase of inequalities; therefore, the ongoing PrevHPV project [12] is evaluating the effect of co-constructed lesson material on HPV vaccine uptake, attitudes, and knowledge, using material that was designed based on adolescents’ preferences around vaccination [29].

Our study has several limitations. Firstly, a self-administered questionnaire among adolescents is at risk of low-quality answers given haphazardly or too quickly, particularly if respondents lacked interest or did not understand the question. We tried to minimise these effects by offering a ‘do not know’ answer modality. We did not analyse durations of questionnaire administrations, but teachers or school nurses were present and surveyed questionnaire administration, which should guarantee a minimal level of information quality. Additionally, the use of anonymous self-administration should have limited the risk of social desirability bias.

Secondly, our participants were not a representative sample of middle school students in France, given the fact that participation depended on the school directors’ agreement and on parental non-opposition, which may have excluded students with parents with strongly negative attitudes towards vaccination. Prevalence estimates should therefore be interpreted with caution. Nevertheless, the HPV vaccine prevalence of 36% among girls and 9% among boys is close to the national estimates for 2021 (37% and 6%). Across all study participants, 21% reported a multilingual background, which corresponds to national survey data from the French Ministry of Culture [30]. Furthermore, our sample included adolescents from a wide range of socio-demographic and socio-economic backgrounds, urban and rural areas and several regions, which allowed an analysis of determinants and should allow a reasonably good generalisability of the identified determinants to the adolescent population in France. Although our study included a relatively large sample, the small sample size in specific subgroups did limit some stratified analyses.

Finally, the requirement of parental consent to vaccination in France could have impacted in a differential way the vaccine intention expressed by adolescents in different socio-educational subgroups.

## Conclusion

Coverage of HPV vaccination in France has been steadily increasing over the last decade, but larger efforts are required to meet programmatic objectives. Of particular importance will be addressing the social inequalities in vaccine awareness, intention to get vaccinated and uptake, demonstrated throughout this study, that could lead to substantial inequalities in HPV-related cancer risk. The adolescents contribute to making decisions on vaccine uptake and our data suggest that policies facilitating and harmonising HPV vaccine promotion by healthcare professionals and adapted school lessons on HPV vaccination could mitigate inequalities in vaccine coverage. A first nation-wide vaccination campaign for 12–13-year-olds is scheduled in France during the current school year 2023-2024, and there is hope that this will not only increase coverage rates, but also reduce related social inequalities.

## Supplementary Data

1172000 Supplementary Material
