# Author's correction for Euro Surveill. 2023;28(46)

**DOI:** 10.2807/1560-7917.ES.2026.31.45.201112c

**Published:** 2026-11-12

**Authors:** Eurosurveillance editorial team

**Affiliations:** 1 European Centre for Disease Prevention and Control (ECDC), Stockholm, Sweden

In the article ‘Can physicians and schools mitigate social inequalities in human papillomavirus vaccine awareness, uptake and vaccination intention among adolescents? A cross-sectional study, France, 2021 to 2022’ by Moffroid et al., published on 16 November 2023, the name of Damien Oudin DOGLIONI was corrected to Damien OUDIN DOGLIONI. This change was made on 12 November 2026 at the request of that author.

