# Supplementary material for: Can physicians and schools mitigate social inequalities in human papillomavirus vaccine awareness, uptake and vaccination intention among adolescents? A cross-sectional study, France, 2021 to 2022
Source: Euro Surveill. 2023 Nov 16;28(46):2300166. doi: 10.2807/1560-7917.ES.2023.28.46.2300166 (PMC10655205; doi:10.2807/1560-7917.ES.2023.28.46.2300166)
Supplement: Supplementary Material [file 23-00166_MUELLER_Supplement.pdf]

Supplementary Material

"This supplementary material is hosted by *Eurosurveillance* as supporting information alongside the article [**Can physicians and schools mitigate social inequalities in human papillomavirus vaccine awareness, uptake and vaccination intention among adolescents? A cross-sectional study, France, 2021-2022**], on behalf of the authors, who remain responsible for the accuracy and appropriateness of the content. The same standards for ethics, copyright, attributions and permissions as for the article apply. Supplements are not edited by *Eurosurveillance* and the journal is not responsible for the maintenance of any links or email addresses provided therein."

SM-1-Table. Key questionnaire items

Shorthand	French question	English Question	Answering Schematic	Coded Schematic
**Sociodemographic variables**				
School year	Vous êtes en?	Which grade are you in?	1. 4ème<br>2. 3ème	/
Sex	Vous êtes?	You are?	1. Girl<br>2. Boy	/
Parental Education Level	Quel est le niveau d'étude de votre mère/ père (ou votre responsable légaux) ? (Si vous avez été élevé(e) principalement par des parents adoptifs, des beaux- parents ou autres, répondez pour ceux avec qui vous avez le plus vécu.)	What is the level of education of your mother and father (or your legal guardians)? (If you were principally raised by adoptive parents, in-law parents or other, respond for those you lived with the longest	1. Inferior to baccalauréat<br>2. Baccalauréat<br>3. Superior to baccalauréat<br>4. I do not know<br>5. Does not apply parents dead, unknown, lost, etc.,	1+2: ≤Bac<br>3: >Bac<br>4: DNK
Multilingualism	Parlez-vous couramment une autre langue que le français avec vos parents?	Do you fluently speak in a language other than French with your parents?	1. Yes (also) another language<br>2. No, just French	1: Multilingual<br>2: French
**Health-related behaviour and aptitudes**				
Self-efficacy	Si l'on vous pose des questions concernant votre santé, par exemple : "As-tu déjà été essoufflé(e) sans faire d'effort ?", "As-tu des allergies ?", "As-tu été opéré(e) des amygdales ?" "Connais-tu le nom de ton médecin généraliste ?". A quel point vous sentez-vous capable d'y répondre sans	If we asked you questions regarding your health, for example: “Have you ever been short of breath without exerting yourself? Do you have any allergies? Have you had your tonsils removed? Do you know the name of your doctor?	Score ranging from 0 - 100	1. 0 – 25<br>2. 26 – 50<br>3. 51 – 75<br>4. 76 - 100

	vos parents ? (Double clique sur le curseur pour le déplacer sur l'échelle, de 0 "Pas du tout capable" à 100 "Parfaitement capable", à la position qui correspond le mieux à ce que vous pouvez faire.)	To what point would you feel capable of responding without your parents? (double click on the cursor to move it along the scale, from 0 “not at all capable” to 100 “perfectly capable”, to the position which corresponds best to what you are capable of).		
Easy finding information	Je pense qu’il est facile de trouver des informations claires sur les vaccins contre les HPV.	I think it is easy to find information that is clear on vaccines against HPV.	1. Completely disagree<br>2. Somewhat disagree<br>3. Unsure<br>4. Somewhat agree<br>5. Completely agree	1 + 2: Disagree<br>3: Unsure<br>4 + 5: Agree
Difficult to talk to close family/ friends about HPV vaccination	Il m’est difficile de parler de la vaccination contre HPV avec mes proches.	I find it difficult to talk about vaccination against HPV with close friends and family.	1. Completely disagree<br>2. Somewhat disagree<br>3. Unsure<br>4. Somewhat agree<br>5. Completely agree	1 + 2: Disagree<br>3: Unsure<br>4 + 5: Agree
Difficult to talk to health professionals about HPV vaccination	Il m'est difficile de parler de la vaccination contre les HPV avec un professionnel de santé (médecin, inffermier, etc.).	I find it difficult to talk about vaccination against HPV with health professionals (e.g., doctors, nurses, etc.)	1. Completely disagree<br>2. Somewhat disagree<br>3. Unsure<br>4. Somewhat agree<br>5. Completely agree	1 + 2: Disagree<br>3: Unsure<br>4 + 5: Agree
**Influence of doctor variables**				
Doctor visit in last 12 months	Avez-vous eu une consultation avec votre médecin	Have you had a consultation with your doctor in the last 12 months?	1. Yes<br>2. No<br>3. I do not remember	

	pendant les douze derniers mois?			
Doctor talked about HPV	Votre médecin vous a-t-il déjà parlé des Papillomavirus Humains (HPV)?	Has your doctor ever talked to you about Human Papillomavirus (HPV)?	1. Yes<br>2. No<br>4. I do not remember	
Doctor offered vaccine	Votre médecin vous a-t-il déjà propose de faire le vaccin contres les HPV?	Has your doctor ever offered you the vaccine against HPV?	1. Yes<br>2. No<br>3. I do not remember	
**Social influence variables**				
Familial Vaccination Attitudes	D'une manière Générale, votre entourage familial est-il favorable à la vaccination?	Generally, would you say your familial entourage is favourable towards vaccinations?	1. Completely unfavourable<br>2. Unfavourable<br>3. Opinions for and against are represented equally<br>4. Favourable<br>5. Completely favourable	1 + 2: Unfavourable<br>3: Unsure<br>4 + 5: Favourable
Social HPV environment	Dans votre entourage, comment décririez vous l'opinion majoritaire sur la vaccination contre les HPV?	In your entourage, how would you describe the majority of the opinions regarding the vaccine against HPV?	1. Completely unfavourable<br>2. Unfavourable<br>3. Opinions for and against are represented equally<br>4. Favourable<br>5. Completely favourable	1 + 2: Unfavourable<br>3: Unsure<br>4 + 5: Favourable
Vaccination status of friends	Avez-vous des ami(e)s qui ont été vacciné(e)s contre les HPV ?	Do you have friends that are vaccinated against HPV?	1. Yes<br>2. No<br>3. Do not know	
**Influence of school curriculum variables**	Au collège, vous a-t-on déjà parlé de:	At school, have they spoken to you about:		
Taught infections are caused by bacteria or viruses	les infections par des virus ou des bactéries?	infections caused by viruses and bacteria?	1. Oui<br>2. No<br>3. Do not remember	2 + 3: No/ DNK<br>1: Yes

Taught what vaccinations are	de la vaccination?	about vaccination?	1. Oui<br>2. No<br>3. Do not remember	2 + 3: No/ DNK<br>1: Yes
Taught human reproduction	la reproduction humaine?	human reproduction?	1. Oui<br>2. No<br>3. Do not remember	2 + 3: No/ DNK<br>1: Yes
Taught sexual education	Au collège, vous a-t-on déjà parlé de l'éducation à la sexualité?	sexual education?	1. Oui<br>2. No<br>3. Do not remember	2 + 3: No/ DNK<br>1: Yes
Taught STIs	les infections sexuellement transmissibles	sexually transmissible infections?	1. Oui<br>2. No<br>3. Do not remember	2 + 3: No/ DNK<br>1: Yes

SM-2-Figure. Flowchart of participant inclusion.

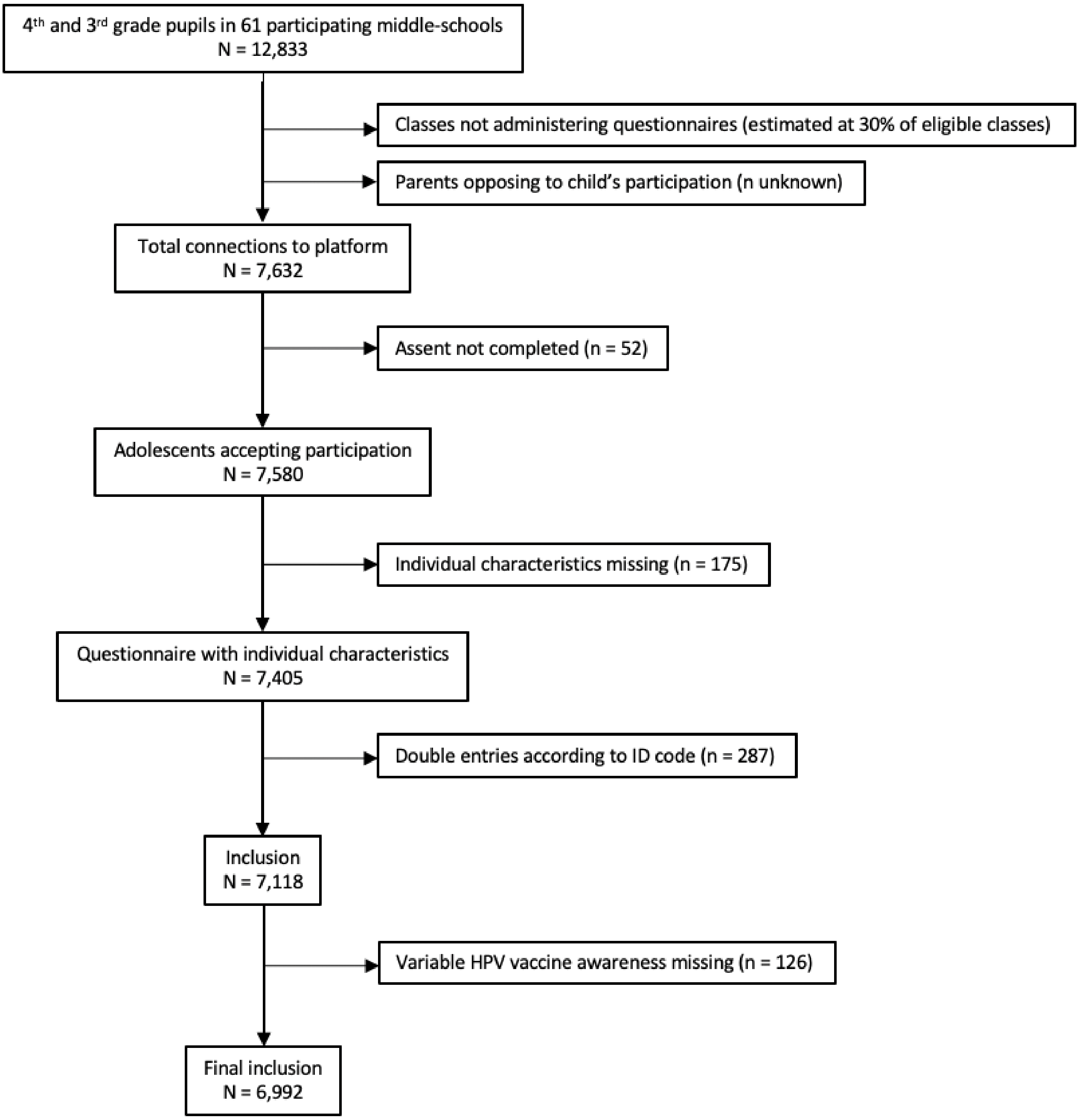

SM-3-Table. Participant characteristics (overall and by sex) among included middle-school pupils in France, 20221-22 (N=6992).

Characteristics	Total (N=6992)		Girls (N=3564)		Boys (N=3428)	
	N	Column %	N	Column %	N	Column %
**School year**						
4th	3742	53.5	1893	53.1	1849	53.9
3rd	3250	46.5	1579	46.9	1579	46.1
**Parental education level**						
Mother						
≤Bac	1521	21.8	776	21.8	745	21.7
>Bac	2257	32.3	1189	33.4	1068	31.1
DNK	3214	46.0	1599	44.9	1615	47.1
Father						
≤Bac	1761	25.2	889	24.9	872	25.4
>Bac	1779	25.4	929	26.1	850	24.8
DNK	3452	49.4	1746	49.0	1706	49.8
**Language**						
Multilingual	1480	21.2	782	21.9	698	20.4
French monolingual	5512	78.8	2782	78.1	2730	79.6
**Education*Language**						
≤Bac*French	1122	16.1	559	15.7	563	16.4
>Bac*French	2198	31.4	1157	32.5	1041	30.4
DNK*French	2192	31.4	1066	29.9	1126	32.9
≤Bac*multilingual	335	4.8	187	5.3	148	4.3
>Bac*multilingual	541	7.7	268	7.5	273	8.0
DNK*multilingual	604	8.6	327	9.2	277	8.1
**School area deprivation level**						
Low	611	8.9	314	8.9	297	8.8
Moderate low	2462	35.7	1242	35.3	1220	36.1
Moderate high	2520	36.5	1270	36.1	1250	37.0
High	1310	19.0	697	19.8	613	18.1

DNK, do not know
Bac, baccalaureat (French high school diploma)

SM-4-Table. General practitioner visit, HPV vaccine offer and remembering school curriculum on vaccination among included middle-school pupils in France, 20221-22 (N=6992).

Row percentages (%)		Remembers general practitioner visit during last 12 months			Remembers school curriculum on vaccination
		No	Yes, without offer	Yes, with offer	Yes
Total		24.1	35.7	40.3	71.2
Class	4th (younger)	25.0	36.6	38.5	68.5
	3rd (older)	23.1	34.6	42.3	74.3
Sex	Girls	19.3	27.9	52.7	71.2
	Boys	31.2	47.2	21.6	71.2
Language	French monolingual	28.2	38.1	33.8	69.9
	Multilingual	23.2	35.1	41.8	76.1
Parental education	<Bac	25.1	36.7	38.3	75.5
	≥Bac	15.9	36.2	47.9	70.1
	DKN	33.1	34.4	32.5	70.0
School area deprivation	Low	24.7	31.8	43.6	68.7
	Moderate low	23.3	35.7	41.0	71.3
	Moderate high	24.0	35.8	40.2	69.6
	High	25.3	37.5	37.2	76.1

SM-5-Figure. HPV-vaccine-related outcomes among included middle-school pupils in France, 20221-22 (N=6992).

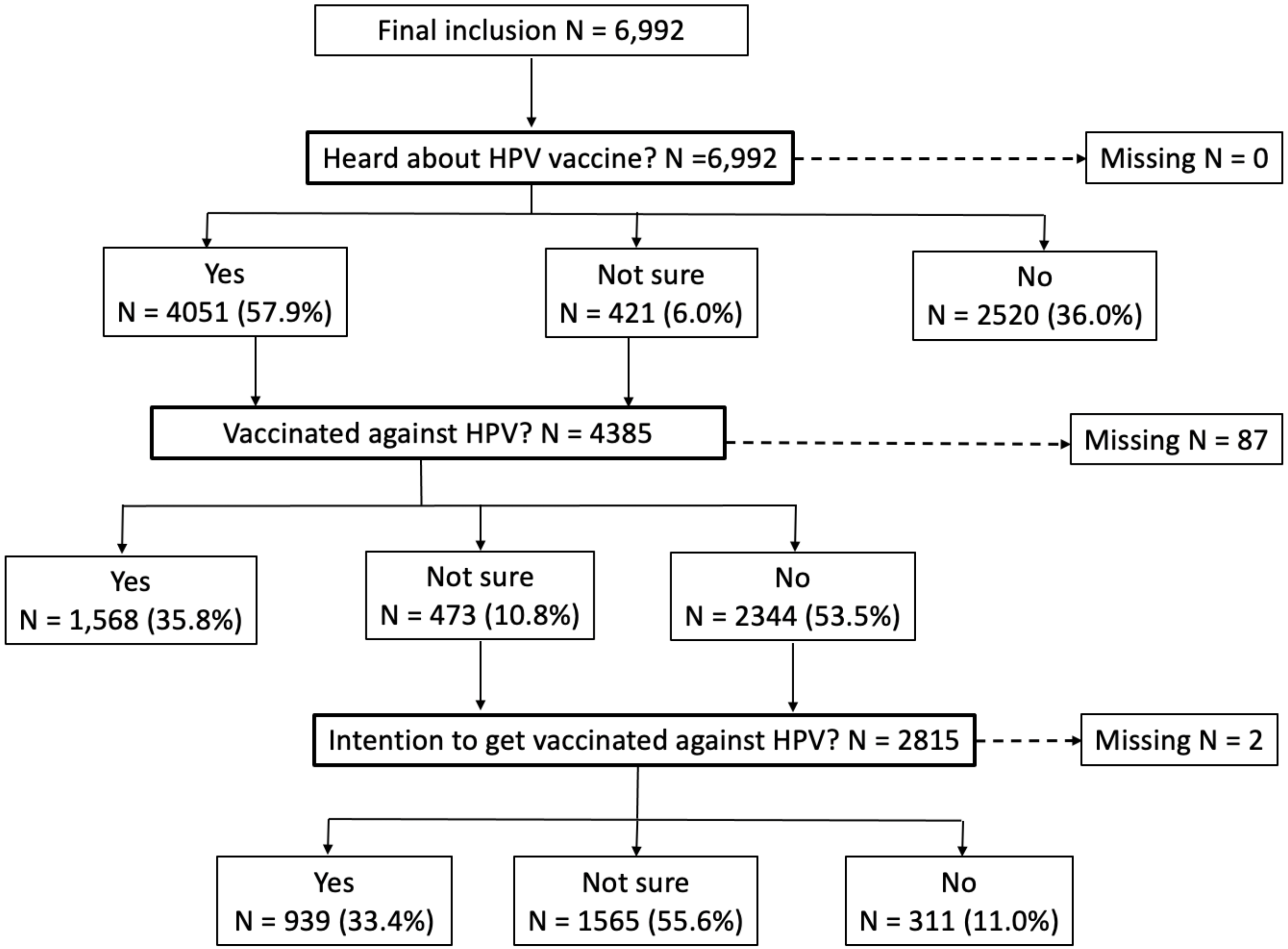

SM-6-Table. Determinants of having heard about HPV and related vaccination among middle-school pupils in France, 20221-22 (N=6992). Bi- and full multivariable logistic regression models.

Characteristics	Having heard of the HPV vaccine				
	Total (column %)	No (row %)	Yes (n = row %)	Bi-variable (p-value)	Multi-variable *(p-value)
	N = 6992	N = 2520 (36)	N = 4472 (64)		
**School year**					
4th	3742 (54)	1430 (38)	2312 (62)	1	1
3rd	3250 (46)	1090 (34)	2160 (66)	1.23 (<0.001)	0.98 (0.704)
**Sex**					
Girl	3564 (51)	860 (24)	2704 (76)	1	1
Boy	3428 (49)	1660 (48)	1768 (52)	0.34 (<0.001)	0.32 (<0.001)
**Parental education level**					
Mum					
≤Bac	1521 (22)	509 (33)	1012 (67)	1	- a
>Bac	2257 (32)	614 (27)	1643 (73)	1.35 (<0.001)	
DNK	3214 (46)	1397 (43)	1817 (57)	0.65 (<0.001)	
Dad					
≤Bac	1761 (25)	563 (32)	1198 (68)	1	- a
>Bac	1779 (25)	505 (28)	1274 (72)	1.19 (0.020)	
DNK	3452 (49)	1452 (42)	2000 (58)	0.65 (<0.001)	
**Language**					
Multilingual	1480 (21)	667 (45)	813 (55)	1	- a
French monolingual	5512 (79)	1853 (34)	3659 (66)	1.62 (<0.001)	
**Education*Language**					
≤Bac*French	1122 (16)	439 (31)	773 (69)	1	1
>Bac*French	2198 (31)	571 (26)	1627 (74)	1.29 (0.002)	1.15 (0.119)
DNK*French	2192 (31)	933 (43)	1259 (57)	0.61 (<0.001)	0.65 (<0.001)
≤Bac*multilingual	335 (5)	143 (43)	192 (57)	0.61 (<0.001)	0.57 (<0.001)
>Bac*multilingual	541 (8)	225 (42)	316 (58)	0.63 (<0.001)	0.55 (<0.001)

DNK*multilingual	604 (9)	299 (49.5)	305 (50.5)	0.46 (<0.001)	0.43 (<0.001)
**School area deprivation level**					
Low	611 (9)	169 (28)	442 (72)	1	1
Moderate low	2462 (36)	944 (38)	1518 (62)	0.61 (<0.001)	0.62 (<0.001)
Moderate high	2520 (37)	833 (33)	1687 (67)	0.77 (0.010)	0.84 (0.116)
High	1310 (19)	541 (41)	769 (59)	0.54 (<0.001)	0.56 (<0.001)
**Health-related behaviour and aptitudes**					
Self-efficacy **					
0-2.5	247 (4)	135 (55)	112 (45)	1	1
2.6-5.0	757 (11)	357 (47)	400 (53)	1.35 (0.041)	1.22 (0.244)
5.1-7.5	1363 (19)	550 (40)	813 (60)	1.78 (<0.001)	1.49 (0.014)
7.6-10	4625 (66)	1478 (32)	3147 (68)	2.57 (<0.001)	1.84 (<0.001)
Easy finding information					
Disagree	334 (7)	23 (7)	311 (93)	1	- a
Unsure	2071 (45)	166 (8)	1905 (92)	0.85 (0.477)	
Agree	2226 (48)	118 (5)	2108 (95)	1.32 (0.238)	
**Influence of doctor**					
Physician visit in last 12 months					
No visit	1233 (18)	588 (48)	645 (52)	1	1
Don't Remember	1030 (15)	409 (40)	621 (60)	1.38 (<0.001)	1.43 (<0.001)
Visited	4598 (67)	1479 (32)	3119 (68)	1.92 (<0.001)	1.60 (<0.001)
**Social influence**					
Familial general vaccination attitudes					
Unfavourable	705 (10)	328 (47)	377 (53)	1	1
Unsure	1547 (22)	627 (41)	920 (59)	1.28 (0.008)	0.98 (0.848)
Favourable	4740 (68)	1565 (33)	3175 (67)	1.77 (<0.001)	1.24 (0.020)
**Influence of school curriculum**					
Infections by bacteria or viruses					
No/ DNK	2482 (36)	1021 (41)	1461 (59)	1	1
Yes	4417 (64)	1477 (33)	2940 (67)	1.39 (<0.001)	1.09 (0.158)

Vaccinations in general					
No/ DNK	1989 (29)	858 (43)	1131 (57)	1	1
Yes	4914 (71)	1632 (33)	3283 (67)	1.53 (<0.001)	1.51 (<0.001)
Human reproduction					
No/ DNK	1135 (16)	463 (41)	672 (59)	1	1
Yes	5766 (84)	2015 (35)	3751 (65)	1.28 (<0.001)	0.88 (0.139)
Sexual education					
No/ DNK	2834 (41)	1086 (41)	1748 (59)	1	1
Yes	4038 (59)	1386 (35)	2652 (65)	1.19 (<0.001)	1.01 (0.906)
Sexually transmitted infections					
No/ DNK	2231 (33)	973 (44)	1258 (56)	1	1
Yes	4615 (67)	1489 (32)	3126 (68)	1.62 (<0.001)	1.50 (<0.001)

DKN, does not know

a In the multivariable model, a combined variable parental education level * language spoken at home was included.

* Multivariable models containing all variables that demonstrated stat sign (P<0.2) in bivariable analyses

** self-efficacy was evaluated on a 10-point scale

SM-7-Table. Determinants of HPV vaccination status among middle-school pupils in France, 20221-22, who have heard about HPV vaccination (N=4385). Bi- and full multivariable logistic regression models.

Characteristics	HPV Vaccination Status				
	Total (n = %)<br>N = 4385	No (n = %)<br>N = 2817 (64.2)	Yes (n = %)<br>N = 1568 (35.8)	Bi-variable (p-value)	Multi-variable * (p-value)
**Schoolyear**					
4th	2271 (52)	1522 (67)	749 (33)	1	1
3rd	2114 (48)	1295 (61)	819 (39)	1.29 (<0.001)	1.24 (0.032)
**Sex**					
Girl	2665 (61)	1390 (52)	1275 (48)	1	1
Boy	1720 (39)	1427 (83)	293 (17)	0.22 (<0.001)	0.42 (<0.001)
**Parental education level**					
<u>Mum</u>					
≤Bac	988 (230	664 (67)	324 (33)	1	- a
>Bac	1615 (37)	907 (56)	708 (44)	1.60 (<0.001)	
DNK	1782 (410	1246 (70)	536 (30)	0.88 (0.139)	
<u>Dad</u>					
≤Bac	1178 (27)	783 (66)	395 (34)	1	- a
>Bac	1245 (28)	703 (56)	542 (44)	1.53 (<0.001)	
DNK	1962 (45)	1331 (68)	631 (32)	0.94 (0.428)	
**Language**					
Multilingual	793 (18)	576 (73)	217 (27)	1	- a
French monolingual	3592 (82)	2241 (62)	1351 (38)	1.60 (<0.001)	
**Education/ Language**					
≤Bac*French	760 (17)	510 (67)	250 (33)	1	1
>Bac*French	1597 (36)	890 (56)	707 (44)	0.62 (<0.001)	1.35 (0.022)
DNK*French	1235 (28)	841 (68)	394 (32)	0.96 (0.645)	1.31 (0.056)
≤Bac*multilingual	185 (4)	139 (75)	46 (25)	0.68 (0.035)	0.75 (0.292)

>Bac*multilingual	309 (7)	206 (67)	103 (33)	1.02 (0.890)	1.39 (0.113)
DNK*multilingual	299 (7)	231 (77)	68 (23)	0.60 (0.001)	0.92 (0.707)
**School area deprivation index**					
Low	438 (10)	257 (59)	181 (41)	1	1
Low/ Moderate	1474 (34)	949 (64)	525 (36)	0.76 (0.030)	0.84 (0.275)
Moderate/ High	1669 (39)	1091 (65)	578 (35)	0.75 (0.010)	0.75 (0.090)
High	752 (17)	487 (65)	265 (35)	0.77 (0.037)	1.03 (0.883)
**Health-related behaviour and aptitudes**					
Self-efficacy **					
0-2.5	106 (2)	70 (66)	36 (34)	1	- [a]
2.6-5.0	391 (9)	274 (70)	117 (30)	0.83 (0.425)	
5.1-7.5	799 (18)	554 (69)	245 (31)	0.86 (0.491)	
7.6-10	3089 (70)	1919 (62)	1170 (38)	1.19 (0.414)	
Difficult to talk to close family/ friends about HPV vaccination					
Agree	592 (14)	440 (74)	152 (26)	1	1
Unsure	1100 (25)	803 (73)	297 (27)	1.07 (0.556)	1.28 (0.149)
Disagree	2693 (61)	1574 (58)	1119 (42)	2.06 (<0.001)	1.04 (0.786)
Difficult to talk to health professional about HPV vaccination					
Agree	580 (13)	412 (71)	168 (29)	1	1
Unsure	1250 (29)	892 (71)	358 (29)	0.98 (0.886)	1.38 (0.050)
Disagree	2555 (58)	1513 (59)	1042 (41)	1.69 (<0.001)	1.21 (0.199)
Easy finding information					
Disagree	302 (7)	215 (71)	87 (29)	1	1
Unsure	1860 (44)	1247 (67)	613 (33)	1.21 (0.153)	0.81 (0.265)
Agree	2079 (49)	1253 (60)	826 (40)	1.63 (<0.001)	0.76 (0.146)
**Influence of doctor**					
No visit	1002 (23)	923 (92)	79 (8)	1	1
Visit, not offered	1520 (35)	1339 (88)	231 (12)	1.21 (0.153)	1.47 (0.045)

Visit and offered	1860 (44)	552 (30)	1259 (70)	27.68 (<0.001)	19.09 (<0.001)
**Social influence**					
Familial Vaccination Attitudes					
Unfavourable	369 (8)	291 (79)	78 (21)	1	1
Unsure	897 (20)	666 (74)	231 (26)	1.29 (0.083)	0.66 (0.045)
Favourable	3119 (71)	1860 (60)	1259 (40)	2.53 (<0.001)	0.90 (0.562)
Social HPV vaccination environment					
Unfavourable	427 (10)	356 (83)	71 (17)	1	1
Unsure	1342 (33)	1057 (79)	285 (21)	1.35 (0.039)	1.80 (0.002)
Favourable	2345 (57)	1189 (51)	1156 (49)	4.87 (<0.001)	3.73 (<0.001)
Vaccination status of friends					
Not vaccinated	446 (10)	382 (86)	64 (14)	1	1
DNK	1642 (38)	1270 (77)	372 (23)	1.75 (<0.001)	1.78 (0.002)
Vaccinated	2218 (52)	1112 (50)	1106 (49)	5.94 (<0.001)	2.97 (<0.001)
**School curriculum**					
Infections by bacteria or viruses					
No/ DNK	1426 (33)	907 (64)	519 (36)	1	- a
Yes	2889 (67)	1862 (64)	1027 (36)	0.96 (0.585)	
Vaccinations in general					
No/ DNK	1108 (26)	685 (62)	423 (38)	1	1
Yes	3220 (74)	2090 (65)	1130 (35)	0.88 (0.065)	0.87 (0.204)
Human reproduction					
No/ DNK	655 (15)	434 (66)	221 (34)	1	- a
Yes	3682 (85)	2347 (64)	1335 (36)	1.13 (0.216)	
Sexual education					
No/ DNK	1715 (40)	1132 (66)	583 (34)	1	1
Yes	2602 (60)	1640 (63)	962 (37)	1.14 (0.046)	1.03 (0.991)
Sexually transmitted infections					
No/ DNK	1233 (29)	822 (67)	411 (33)	1	1
Yes	3067 (71)	1940 (63)	1127 (37)	1.16 (0.035)	1.03 (0.810)

DKN, does not know
a In the multivariable model, a combined variable parental education level * language spoken at home was included.

* Multivariable models containing all variables that demonstrated stat sign (P<0.2) in bivariable analyses
** self-efficacy was evaluated on a 10-point scale

SM-8-Table. Determinants of intention to get vaccinated with the HPV vaccine among HPV unvaccinated middle-school pupils in France, 2021-22 (N=2815). Bi- and full multivariable logistic regression models.

Characteristics	Intention to get vaccinated with the HPV vaccine							
	Total (n, %)	Refusal (n, %)	Indecision (n, %)	Intention (n, %)	Bi-variable (p-value)		Multi-variable (p-value)	
	N = 2815	N = 311 (11)	N = 1565 (56)	N = 939 (33)	Indecision vs Refusal	Intention vs Refusal	Indecision vs Refusal	Intention vs Refusal
**Schoolyear**								
4th	1521 (54)	179 (12)	853 (56)	489 (32)	1	1	1	1
3rd	1294 (46)	132 (10)	712 (55)	450 (35)	1.13 (0.323)	1.25 (0.093)	1.08 (0.628)	1.17 (0.372)
**Sex**								
Girl	1388 (49)	124 (9)	735 (53)	529 (38)	1	1	1	1
Boy	1427 (51)	187 (13)	830 (58)	410 (29)	0.75 (0.022)	0.51 (<0.001)	0.61 (0.001)	0.47 (<0.001)
**Parental education level**								
<u>Mum</u>								
≤Bac	664 (24)	78 (12)	356 (54)	230 (35)	1	1	- a	- a
>Bac	906 (32)	76 (8)	466 (51)	364 (40)	1.34 (0.093)	1.62 (0.008)		
DNK	1245 (44)	157 (13)	743 (60)	345 (28)	1.04 (0.813)	0.75 (0.071)		
<u>Dad</u>								
≤Bac	782 (28)	91 (12)	399 (51)	292 (37)	1	1	- a	- a
>Bac	703 (25)	51 (7)	393 (56)	259 (37)	1.76 (0.003)	1.58 (0.018)		
DNK	1330 (47)	169 (13)	773 (58)	388 (29)	1.04 (00769)	0.72 (0.027)		
**Language**								
Multilingual	575 (20)	91 (16)	337 (59)	147 (26)	1	1	- a	- a
French monolingual	2240 (80)	220 (10)	1228 (55)	792 (35)	1.51 (0.003)	2.23 (<0.001)		
**Education/ Language**								
≤Bac*French	510 (18)	62 (12)	262 (51)	186 (20)	1	1	1	1
>Bac*French	889 (32)	57 (6)	464 (52)	368 (39)	1.93 (0.001)	2.15 (<0.001)	1.53 (0.071)	1.47 (0.134)
DNK*French	841 (30)	101 (12)	502 (60)	238 (25)	1.18 (0.363)	0.78 (0.201)	0.98 (0.923)	0.72 (0.179)

≤Bac*multilingual	139 (5)	22 (16)	74 (53)	43 (31)	0.80 (0.417)	0.65 (0.154)	0.96 (0.906)	0.95 (0.884)
>Bac*multilingual	206 (7)	30 (15)	120 (58)	56 (27)	0.95 (0.825)	0.62 (0.078)	0.94 (0.843)	0.51 (0.053)
DNK*multilingual	230 (8)	39 (17)	143 (62)	48 (21)	0.87 (0.536)	0.41 (0.001)	0.82 (0.497)	0.45 (0.019)
**School area deprivation index**								
Low	257 (9)	23 (9)	128 (50)	106 (41)	1	1	1	1
Low/ Moderate	949 (34)	103 (11)	541 (57)	305 (32)	0.94 (0.818)	0.64 (0085)	1.06 (0.850)	0.72 (0.317)
Moderate/ High	1090 (39)	109 (10)	598 (55)	383 (35)	0.99 (0.954)	0.76 (0.286)	1.13 (0.695)	0.88 (0.692)
High	486 (17)	65 (13)	285 (59)	136 (28)	0.79 (0.368)	0.50 (0.004)	0.73 (0.327)	0.29 (0.042)
**Health-related behaviour and aptitudes**								
Self-efficacy **								
0-2.5	70 (2)	15 (21)	38 (54)	17 (24)	1	1	1	1
2.6-5.0	273 (10)	42 (15)	156 (57)	75 (27)	1.47 (0.276)	1.58 (0.260)	1.78 (0.186)	1.44 (0.496)
5.1-7.5	554 (20)	49 (9)	342 (62)	163 (29)	2.76 (0.0030	2.94 (0.006)	2.41 (0.036)	1.92 (0.202)
7.6-10	1918 (68)	205 (11)	1029 (54)	684 (36)	1.98 (0.030)	2.94 (0.003)	1.74 (0.161)	1.49 (0.407)
Difficult to talk to close family/ friends about HPV vaccination								
Agree	440 (16)	54 (12)	265 (60)	121 (28)	1	1	1	1
Unsure	802 (28)	96 (12)	523 (65)	183 (23)	1.11 (0.574)	0.85 (0.434)	0.95 (0.847)	0.89 (0.680)
Disagree	1573 (56)	161 (10)	777 (49)	635 (40)	0.98 (0.923)	1.76 (0.0020	0.79 (0.295)	1.04 (0.874)
Difficult to talk to health professional about HPV vaccination								
Agree	412 (15)	53 (13)	230 (56)	129 (31)	1	1	1	1
Unsure	892 (32)	113 (13)	573 (64)	206 (23)	1.17 (0.397)	0.75 (0.280)	1.09 (0.729)	0.95 (0.862)
Disagree	1511 (54)	145 (10)	762 (50)	604 (40)	1.21 (0.280)	1.71 (0.004)	1.09 (0.695)	1.21 (0.464)
Easy finding information								
Disagree	215 (8)	65 (30)	91 (42)	59 (27)	1	1	1	1

Unsure	1247 (46)	149 (12)	775 (62)	323 (26)	3.71 (<0.001)	2.39 (<0.001)	2.45 (<0.001)	1.43 (0.178)
Agree	1251 (46)	87 (7)	635 (51)	529 (42)	5.21 (<0.001)	6.70 (<0.001)	3.11 (<0.001)	2.63 (<0.001)
**Influence of doctor**								
No visit	922 (33)	118 (13)	567 (62)	237 (26)	1	1	1	1
Visit, not offered	1338 (48)	157 (12)	800 (60)	381 (28)	1.06 (0.661)	1.21 (0.199)	0.96 (0.800)	0.97 (0.883)
Visit and offered	552 (20)	36 (7)	197 (36)	319 (58)	1.14 (0.531)	4.41 (<0.001)	1.15 (0.567)	3.21 (<0.001)
**Social influence**								
Familial general vaccination Attitudes								
Unfavourable	291 (10)	94 (32)	154 (53)	43 (15)	1	1	1	1
Unsure	666 (24)	84 (13)	443 (67)	139 (21)	3.22 (<0.001)	3.62 (<0.001)	2.07 (0.001)	1.74 (0.052)
Favourable	1858 (66)	133 (7)	968 (52)	757 (41)	4.44 (<0.001)	12.44 (<0.001)	2.39 (<0.001)	3.66 (<0.001)
Social HPV vaccination environment								
Unfavourable	356 (14)	134 (38)	186 (52)	36 (10)	1	1	1	1
Unsure	1057 (41)	108 (10)	731 (69)	218 (21)	4.88 (<0.001)	7.50 (<0.001)	3.62 (<0.001)	7.28 (<0.001)
Favourable	1187 (46)	51 (4)	508 (43)	628 (53)	7.18 (<0.001)	45.83 (<0.001)	5.20 (<0.001)	31.07 (<0.001)
Vaccination status of friends								
Not vaccinated	382 (14)	81 (21)	224 (59)	77 (20)	1	1	1	1
DNK	1270 (46)	129 (10)	826 (65)	315 (25)	2.32 (<0.001)	2.57 (<0.001)	1.87 (0.001)	1.90 (0.006)
Vaccinated	1110 (40)	96 (9)	481 (43)	533 (48)	1.81 (0.0010	5.84 (<0.001)	1.38 (0.109)	3.17 (<0.001)
**Influence of school curriculum**								
Infections by bacteria or viruses								
No/ DNK	905 (33)	107 (12)	513 (57)	285 (31)	1	1	1	1
Yes	1862 (67)	199 (11)	1020 (55)	643 (35)	1.07 (0.612)	1.21 (0.166)	0.88 (0.438)	0.87 (0.454)
Vaccinations in general								
No/ DNK	685 (25)	88 (13)	385 (25)	212 (31)	1	1	1	1
Yes	2088 (75)	217 (10)	1155 (55)	716 (34)	1.22 (0.160)	1.37 (0.034)	1.12 (0.589)	0.94 (0.789)
Human reproduction								

No/ DNK	433 (16)	49 (11)	237 (55)	147 (34)	1	1	-	-
Yes	2346 (84)	259 (11)	1307 (56)	780 (33)	1.04 (0.804)	1.0 (0.983)		
Sexual education								
No/ DNK	1131 (41)	131 (12)	630 (56)	370 (33)	1	1	-	-
Yes	1639 (59)	175 (11)	908 (55)	556 (34)	1.08 (0.549)	1.12 (0.378)		
Sexually transmitted infections								
No/ DNK	821 (30)	89 (11)	465 (57)	267 (33)	1	1	-	-
Yes	1939 (70)	216 (11)	1065 (55)	658 (34)	0.94 (0.674)	1.02 (0.916)		

DKN, does not know

a In the multivariable model, a combined variable parental education level * language spoken at home was included.

** self-efficacy was evaluated on a 10-point scale

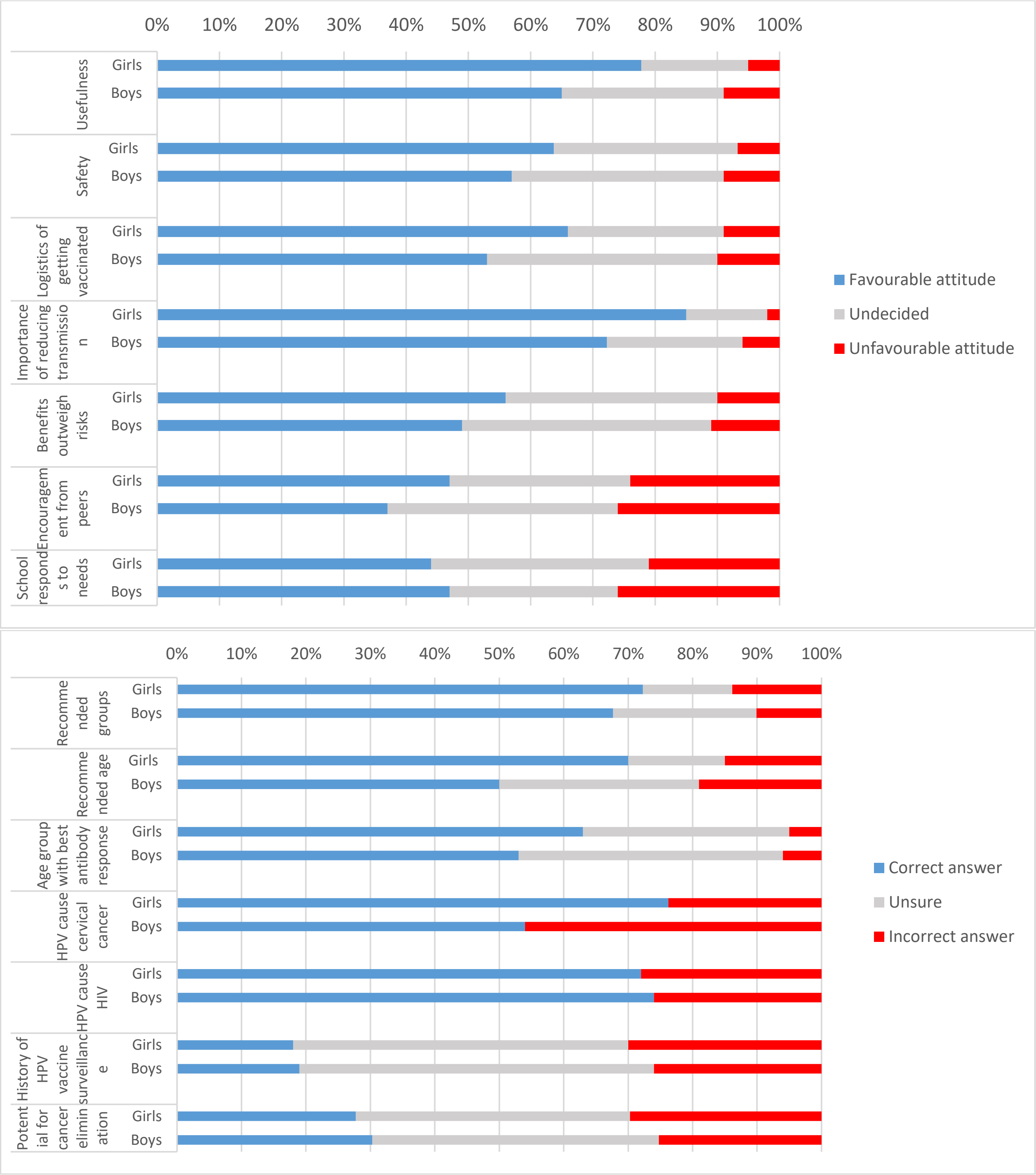

SM-9-Figure. Distribution of attitude (upper panel) and knowledge (lower panel) items surrounding HPV vaccination among middle-school pupils who have heard about HPV vaccination, by sex. France, 20221-22 (N=4,333).

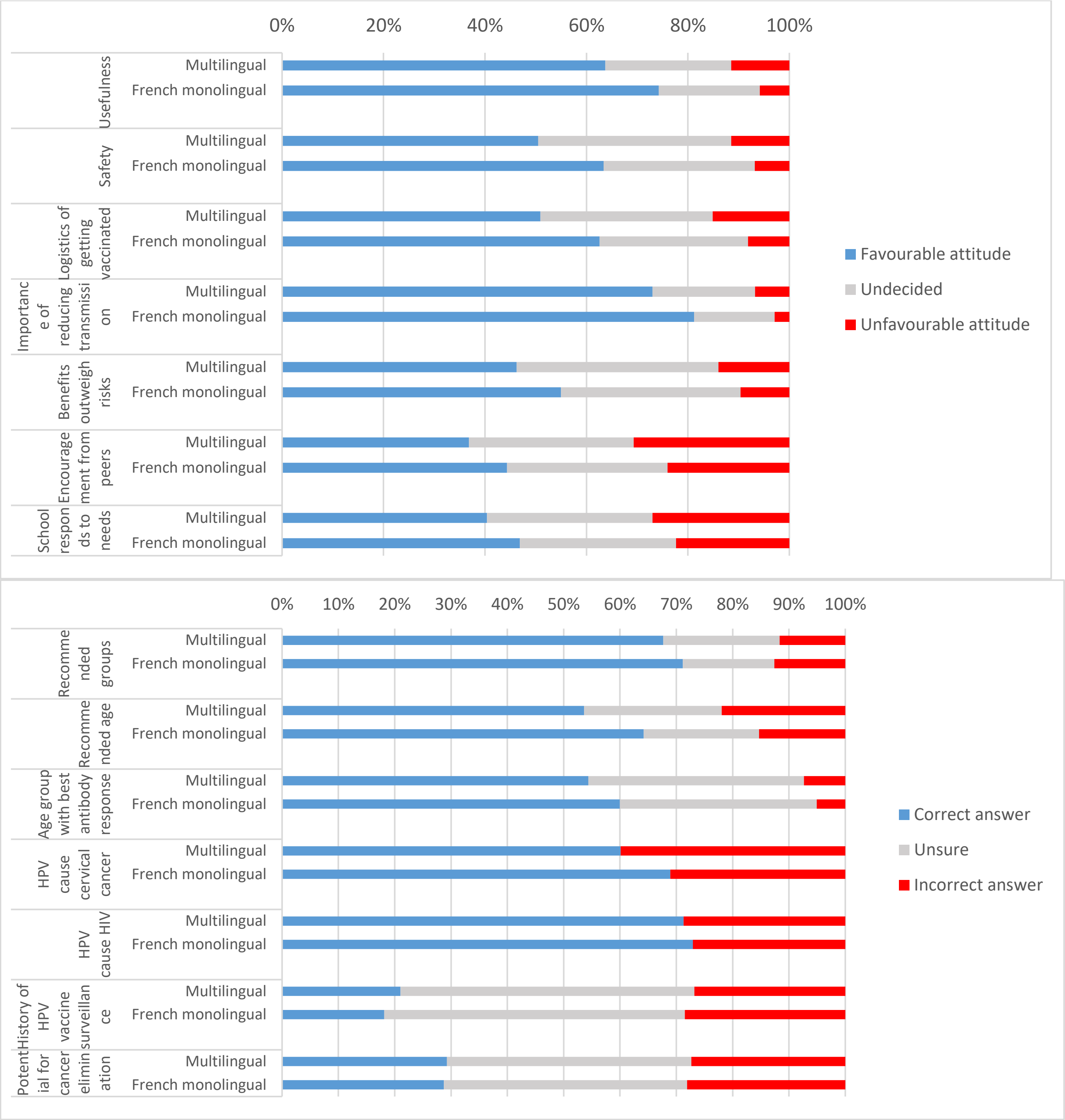

SM-10-Figure. Distribution of attitude (upper panel) and knowledge (lower panel) items surrounding HPV vaccination among middle-school pupils who have heard about HPV vaccination, by multilingualism. France, 20221-22 (N=4,333).

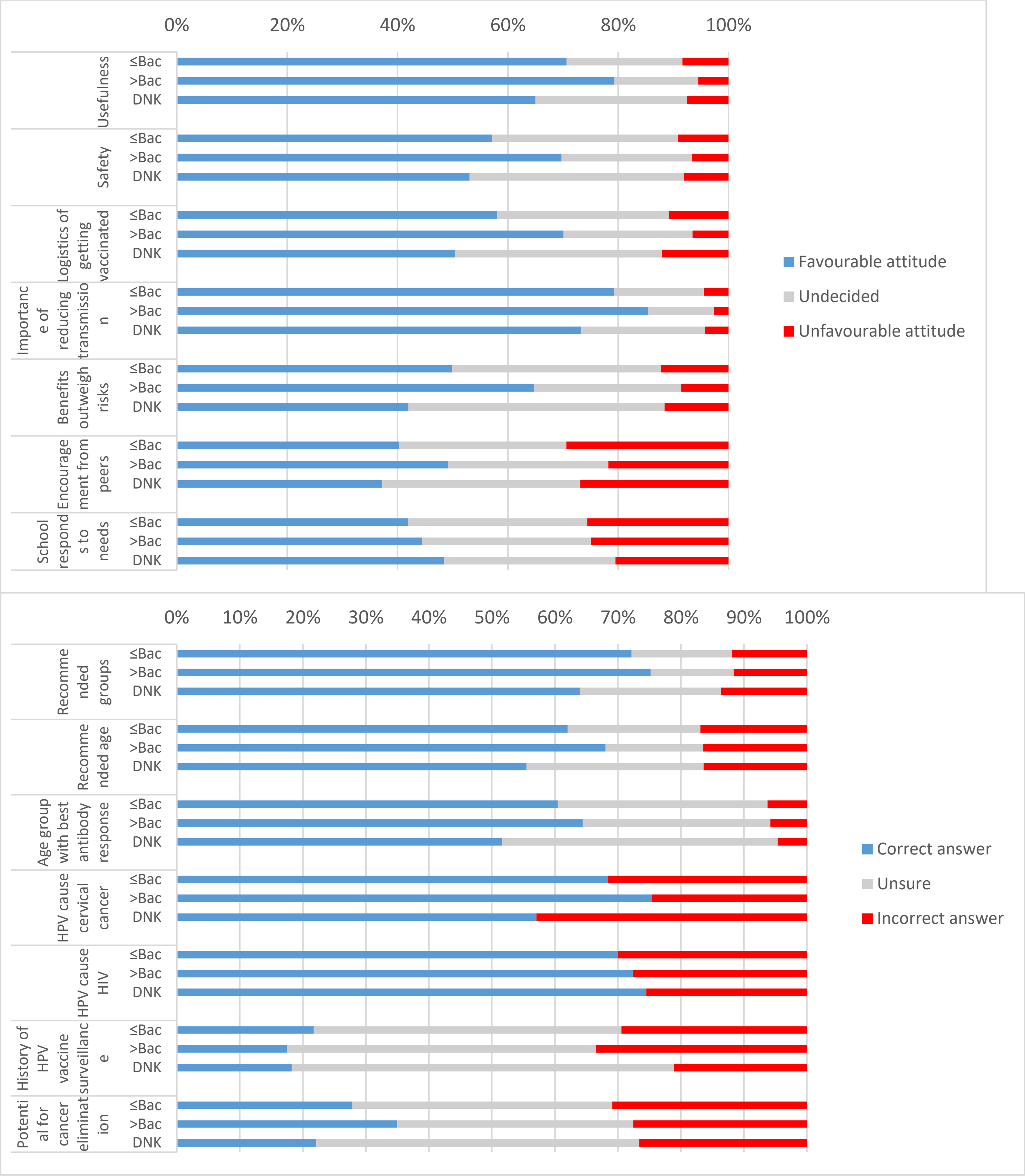

SM-11-Figure. Distribution of attitude (upper panel) and knowledge (lower panel) items surrounding HPV vaccination among middle-school pupils who have heard about HPV vaccination, by parental education. France, 20221-22 (N=4,333).

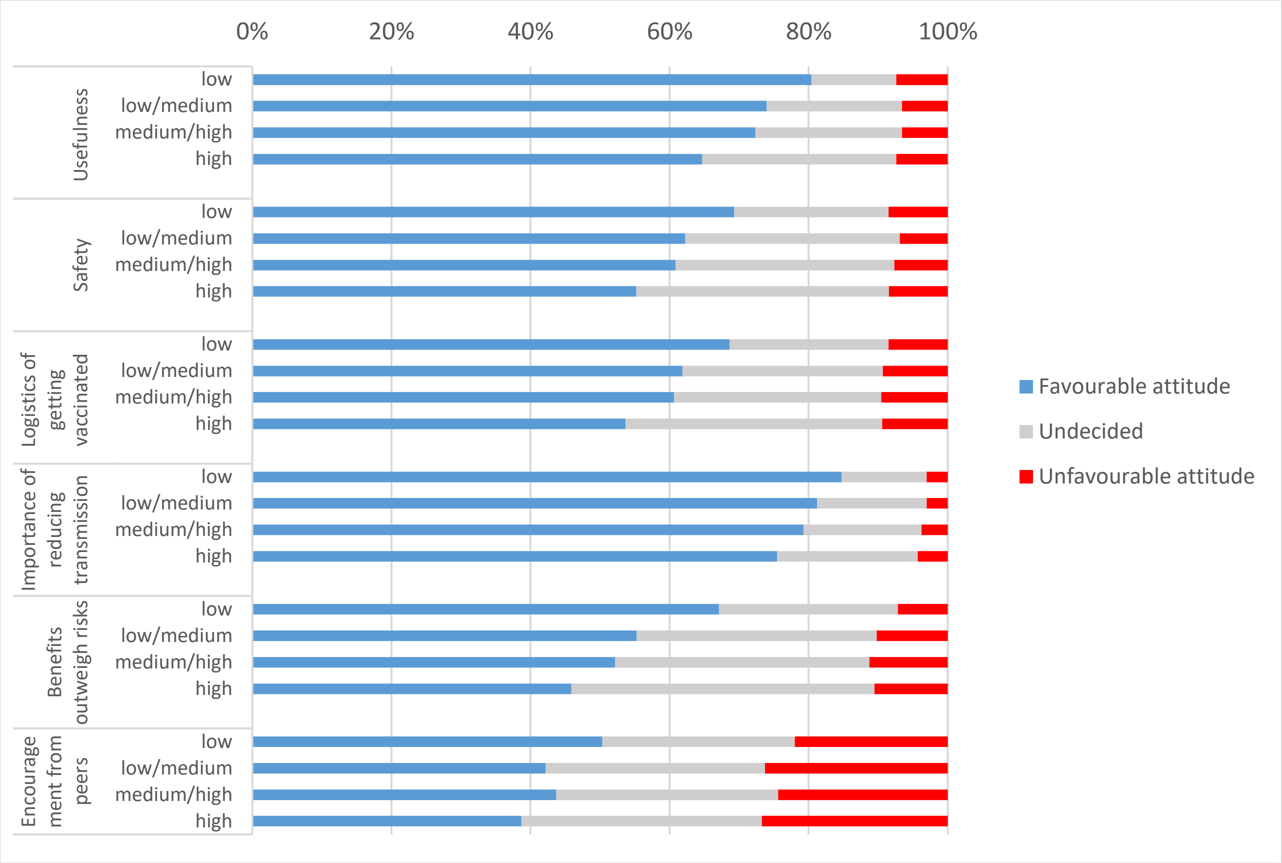
0%
20%
40%
60%
80%
100%
Usefulness
Safety
Logistics of getting vaccinated
Importance of reducing transmission
Benefits outweigh risks
Encourage ment from peers
low
low/medium
medium/high
high
Favourable attitude
Undecided
Unfavourable attitude

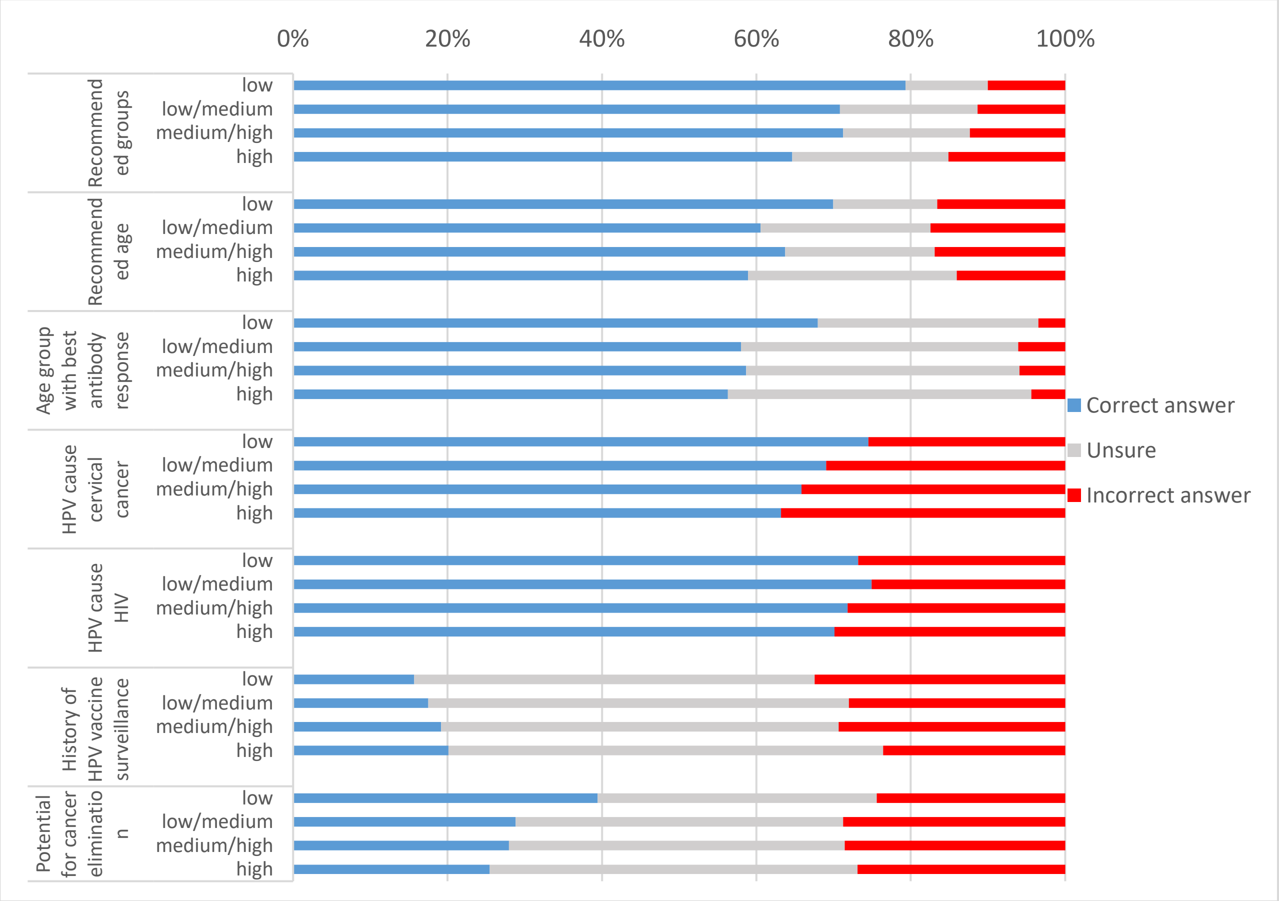
0%
20%
40%
60%
80%
100%
Recommend ed groups
Recommend ed age
Age group with best antibody response
HPV cause cervical cancer
HPV cause HIV
History of HPV vaccine surveillance
Potential for cancer eliminatio n
low
low/medium
medium/high
high
Correct answer
Unsure
Incorrect answer

SM-12-Figure. Distribution of attitude (upper panel) and knowledge (lower panel) items surrounding HPV vaccination among middle-school pupils who have heard about HPV vaccination, by school area deprivation level. France, 20221-22 (N=4,333).

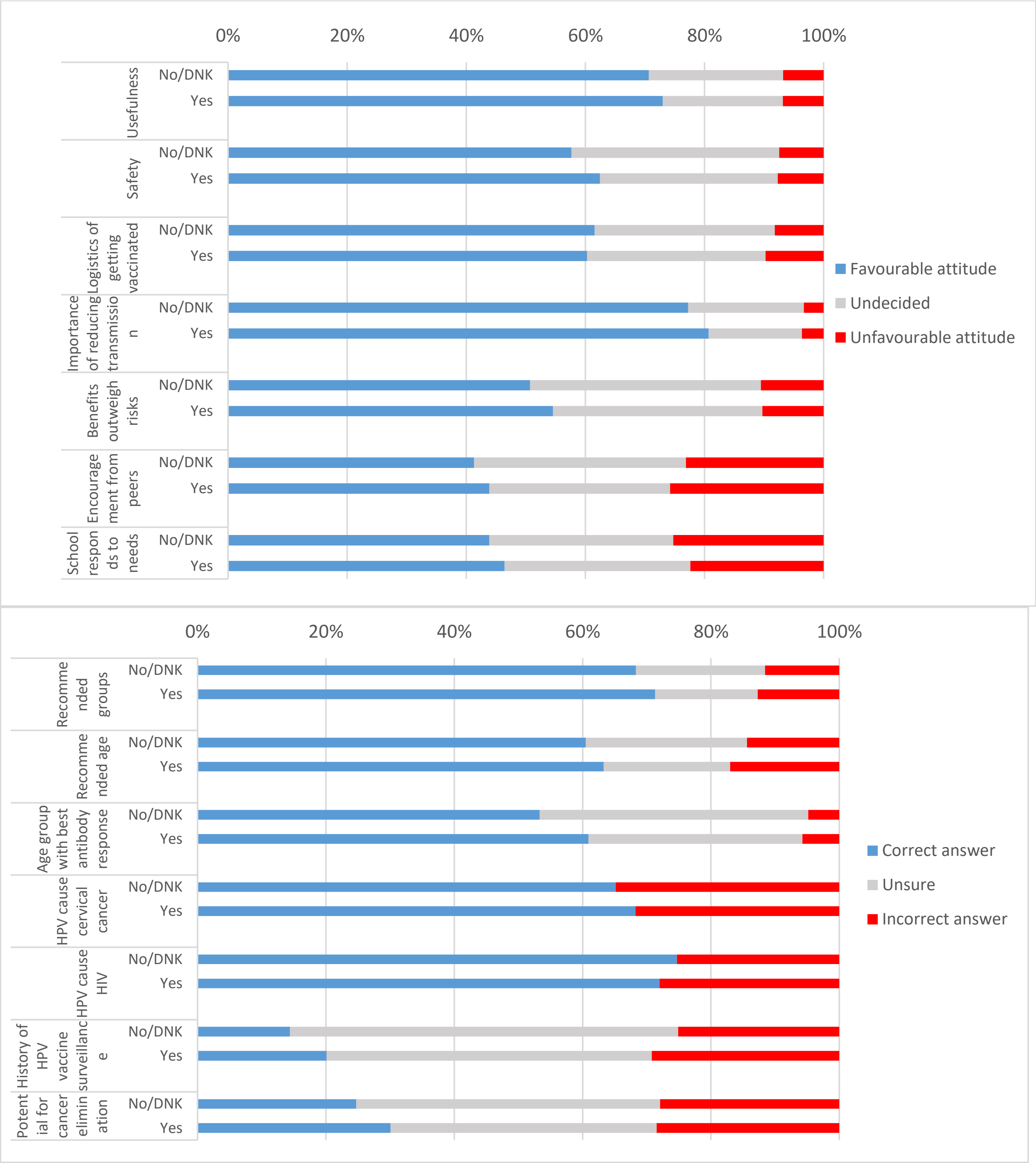

SM-13-Figure. Distribution of attitude (upper panel) and knowledge (lower panel) items surrounding HPV vaccination among middle-school pupils who have heard about HPV vaccination, by remembering school curriculum on vaccination. France, 20221-22 (N=4,333).
